# Snapshot of Viral Infections in Wild Carnivores Reveals Ubiquity of Parvovirus and Susceptibility of Egyptian Mongoose to Feline Panleukopenia Virus

**DOI:** 10.1371/journal.pone.0059399

**Published:** 2013-03-20

**Authors:** Margarida D. Duarte, Ana Margarida Henriques, Sílvia Carla Barros, Teresa Fagulha, Paula Mendonça, Paulo Carvalho, Madalena Monteiro, Miguel Fevereiro, Mafalda P. Basto, Luís Miguel Rosalino, Tânia Barros, Victor Bandeira, Carlos Fonseca, Mónica V. Cunha

**Affiliations:** 1 INIAV, I.P.- Instituto Nacional de Investigação Agrária e Veterinária, Unidade Estratégica de Produção e Saúde Animal, Lisboa, Portugal; 2 Universidade de Lisboa, Centro de Biologia Ambiental, Faculdade de Ciências de Lisboa, Lisboa, Portugal; 3 Laboratório de Ecologia Isotópica/CENA, Universidade de São Paulo, São Paulo, Brasil; 4 Departamento de Biologia and CESAM, Universidade de Aveiro, Aveiro, Portugal; Columbia University, United States of America

## Abstract

The exposure of wild carnivores to viral pathogens, with emphasis on parvovirus (CPV/FPLV), was assessed based on the molecular screening of tissue samples from 128 hunted or accidentally road-killed animals collected in Portugal from 2008 to 2011, including Egyptian mongoose (*Herpestes ichneumon*, n = 99), red fox (*Vulpes vulpes*, n = 19), stone marten (*Martes foina*, n = 3), common genet (*Genetta genetta*, n = 3) and Eurasian badger (*Meles meles*, n = 4). A high prevalence of parvovirus DNA (63%) was detected among all surveyed species, particularly in mongooses (58%) and red foxes (79%), along with the presence of CPV/FPLV circulating antibodies that were identified in 90% of a subset of parvovirus-DNA positive samples. Most specimens were extensively autolysed, restricting macro and microscopic investigations for lesion evaluation. Whenever possible to examine, signs of active disease were not present, supporting the hypothesis that the parvovirus *vp2* gene fragments detected by real-time PCR possibly correspond to viral DNA reminiscent from previous infections. The molecular characterization of viruses, based on the analysis of the complete or partial sequence of the *vp2* gene, allowed typifying three viral strains of mongoose and four red fox’s as feline panleukopenia virus (FPLV) and one stone marten’s as newCPV-2b type. The genetic similarity found between the FPLV viruses from free-ranging and captive wild species originated in Portugal and publicly available comparable sequences, suggests a closer genetic relatedness among FPLV circulating in Portugal.

Although the clinical and epidemiological significance of infection could not be established, this study evidences that exposure of sympatric wild carnivores to parvovirus is common and geographically widespread, potentially carrying a risk to susceptible populations at the wildlife-domestic interface and to threatened species, such as the wildcat (*Felis silvestris*) and the critically endangered Iberian lynx (*Lynx pardinus*).

## Introduction

Canine parvovirus (CPV) and feline panleukopenia virus (FPLV) are closely related viruses that have been included in the unique species *Feline panleukopenia virus* together with other antigenic and genetically related viruses, such as raccoon parvovirus (RPV), raccoon dog parvovirus (RDPV), blue fox parvovirus (BFPV) and mink enteritis virus (MEV) [1], [2], [3]. All together, these viruses infect a wide range of domestic and wild species of the order Carnivora [2]. VP2, the major structural protein of the viral capsid, determines the pathogenicity, tissue tropism and host ranges of this virus subgroup [4], [5]. FPLV was originally identified in domestic cats [6] and later on other large felids, such as tigers, panthers, cheetahs and lions [2], [7], [8], [9], [10], [11], [12]. Canine parvovirus (CPV-2) was detected for the first time in 1978, possibly emerging from a FPLV like-virus [13]. This highly virulent virus rapidly became endemic in dogs throughout the world. Original CPV-2 strain did not infect cats [4], however it was replaced by new antigenic variants, designated CPV-2a, CPV-2b and CPV-2c that regained the ability to infect felids [3],[5],[14].

Depending on age and immunological status, the infection of young domestic carnivores and a few species of large felids can be sub-clinical, acute (characterized by leukopenia, fever, depression, dehydration, and diarrhoea), or cause sudden death [2],[15]. However, in mustelids (otters, badgers, ferrets, martens and fishers) and viverrids (genets and civets), the pathogenicity of the disease caused by feline-like parvoviruses is still unclear. Reports refer mainly to serological or virological evidences rather than to clinical or anatomo-histological data (reviewed by [2]). MEV infection of minks is an exception, since most infected animals, in particular the young ones, develop acute hemorrhagic enteritis, frequently associated with leukopenia [16].

Little information is presently available on the incidence of parvovirus in mesocarnivores from Portugal, but the existing serological [17] and virological studies [18],[19] suggest the exposure of red foxes, common genets and stone martens to infection.

Although the carnivore guild in mainland Portugal is highly diversified, comprising 14 species, among the strictly terrestrial predators, only red fox, stone marten, badger, common genet and, more recently, the Egyptian mongoose, have a known generalized distribution [20],[21]. Genets and mongooses are carnivores whose distribution is mainly restricted in Europe to the Iberian Peninsula (Portugal and Spain) [22],[23]. Only genet occupies southern France territories [23]. Due to several factors, namely the recent abandon of croplands, rural depopulation, great adaptability in terms of its bio-ecology and lack of natural predators, mongoose has been expanding rapidly from South to North, and, recently, it has invaded the Northeastern areas of Portugal from where it was absent in the beginning of the century [21],[24]. The population biology of these animal species is still largely unknown, namely their contribution to pathogen cross-species transmission. Therefore, the aim of the present work was to refine our understanding of viruses circulating in the wild. For this purpose, we determined the incidence of particular viruses in the most widely distributed, strictly terrestrial, wild carnivore species free-ranging in mainland Portugal: Egyptian mongoose, red fox, stone marten, Eurasian badger, and common genet, obtained from road-kills or harvested during predator control actions (mongoose and fox). Furthermore, the knowledge on the molecular properties of field parvoviruses that circulate within these populations was extended based on sequence analysis. Even though animal sampling was widespread, including sensitive areas for conservation, it was more intense in the South region of the mainland, next to priority intervention areas of the Iberian lynx Action Plan in Portugal that potentially offer suitable habitat for the reintroduction of this endangered carnivore species.

## Results

### Preliminary Screening of 34 Specimens for Relevant Viral Pathogens Evidenced the Presence of Parvovirus in Wild Carnivores

In a first stage of the present study, the presence of parvovirus (PV), Coronavirus (CoV), Canine Distemper Virus (CDV), Feline Herpesvirus (FHV), Aujeszky Disease virus (ADV), Canine Adenovirus types 1 and 2 (CAV1 and CAV2) and Influenza A virus (IV) was investigated on tissue samples by real-time PCR (PV, FHV, ADV, CAV1/CAV2) and by reverse transcription real-time PCR (CoV and IV), on a small scale pilot survey performed on 34 specimens from the Herpestidae, Canidae and Mustelidae families of the order Carnivora (Table 1).

**Table 1:** Results of the virological survey in free-ranging wild carnivores from Portugal.

Wild Carnivore			Virus tested (positive samples/total samples tested)							
Animal Species	No.specimens	CollectionMethod^a^	Parvovirus^b^	PV^c^	CoV^c^	CDV^c^	FHV^c^	ADV^c^	CAV1/CAV2^c^	InfluenzaA^c^
Red fox (*Vulpes vulpes*)	19	R, H	15/19 (78.9%)	4/4	0/4	0/4	0/4	0/4	0/4	0/4
Egyptian mongoose (*Herpestes ichneumon*)	99	R, H	57/99 (57.8%)	14/28	1/28	0/28	0/28	0/28	0/28	0/28
Stone marten (*Martes foina*)	4	R	3/4	1/1	0/1	0/1	0/1	0/1	0/1	0/1
Eurasian badger (*Meles meles*)	3	R	3/3	0/1	0/1	0/1	0/1	0/1	0/1	0/1
Common genet (*Genetta genetta*)	3	R	3/3	–	–	–	–	–	–	–

a specimen origin: Hunting (H) or Road-kill (R).

b Results of the parvovirus survey in 128 specimens.

c Results of the preliminary virological survey in 34 specimens. Parvovirus (PV), Coronavirus (CoV), Canine Distemper Virus (CDV), Feline Herpesvirus (FHV), Aujeszky Disease virus *ADV), Canine Adenovirus types 1 and 2 (CAV1 and CAV2) and Influenza A virus.

Parvovirus DNA was detected in 19 animals, specifically mongoose (n = 14 out of 28, 50%), red fox (n = 4) and stone marten (n = 1) (Table 1). The single Eurasian badger specimen tested at this phase was parvovirus-DNA negative. Only one mongoose, which was also exposed to PV, as indicated by PCR, tested positive for CoV. All animal specimens were negative for CDV, CAV1, CAV2, ADV, Influenza A and FHV (Table 1).

### The High Sensibility of Real-time PCR as Detection Method and Availability of Tissue Matrices Favored Parvovirus DNA Detection, Disclosing Marked Incidences in Mongoose and Red Fox Subpopulations

**Figure 1:**
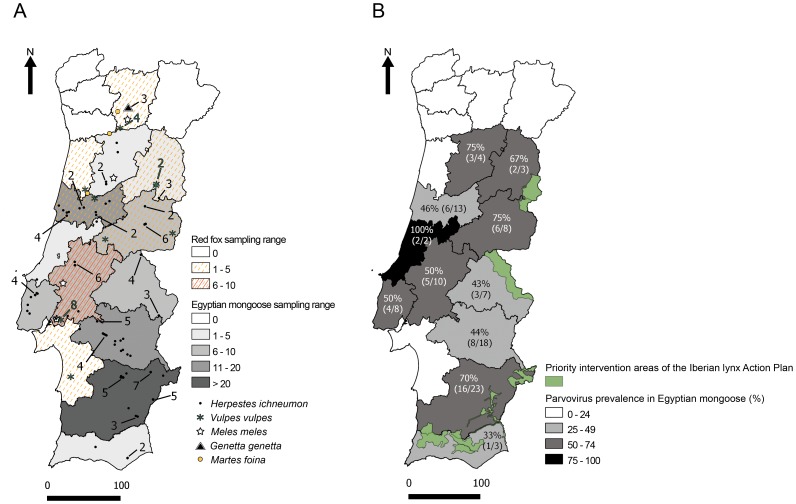
Geographical distribution and sampling range of animal specimens and regional prevalence rates of parvovirus in mongoose. (A) The animal species are represented in the map by symbols, as indicated. The number of samples with the same GPS coordinates (latitude; longitude) is indicated by the Arabic numbers. Thick lines separate Portugal districts. The distribution and range sampling per district of red fox and mongoose subpopulations are evidenced in orange and grey scales, respectively, as indicated. (B) Prevalence of parvovirus in mongoose *per* sampled district; the number of PCR-positive samples per total sampling is indicated in brackets. Priority intervention areas of the Iberian lynx Action Plan are shown in green. Figure produced with open-access software QGIS.

**Figure 2:**
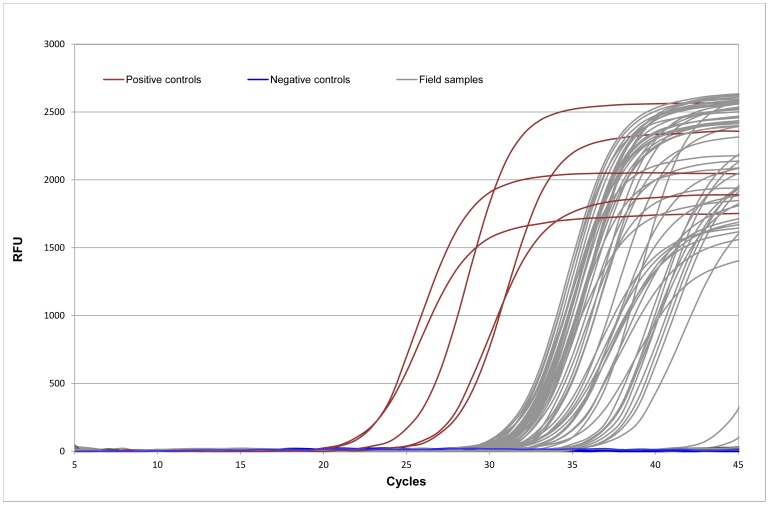
Parvovirus DNA amplification curves obtained during real-time PCR assays with tissue samples from wild carnivores. The relative fluorescence units (RFU) are plotted against cycle number. Ct variation was observed among positive samples from all surveyed species, with the exception of genet tissues, which yielded homogeneous values below 35. The average Ct was 34.73 cycles (standard deviation of 3.36). Values above 40 were considered negative. The Ct values of the positive controls used in each assay, a commercial vaccine containing the old CPV-2 strain, are also represented. Negative controls had no measurable fluorescence indicated by flat lines in the plot.

Since the first subset of animal samples tested negative for the large majority of the viruses screened, only PV was evaluated on a broader sample (Fig. 1A). Parvovirus *vp2* sequences (93 bp) were detected by real-time PCR in lymph node and intestine samples of 81 out of 128 specimens (Fig. 1A, Table 1). The Ct values were high, ranging from 27.92 to 39.75 (Fig. 2). The average Ct was 34.73 cycles (standard deviation, sd, 3.36). Amplification of parvovirus DNA yielded clear sigmoid-shaped, high-fluorescence, amplification curves, while negative controls had no measurable fluorescence indicated by a flat line in the plot (Fig. 2).

The high incidence of PV DNA in sampled specimens (63.3%) suggests that, in Portugal, mesocarnivore species are highly exposed to PV infection (Table 1). Almost 58% of Egyptian mongooses examined (n = 57 out of 99) were PV DNA-positive (Table 1, Fig. 1B). There were positive animals in all sampled districts, however the incidence was higher in the South (70%), if considering the areas with the highest sampling range (Fig. 1B). Despite very restricted sampling, all genets (n = 3) and stone martens (n = 3) tested, as well as three of four badgers examined, were DNA-positive. Also limited in sample size, the red fox subpopulation exhibited the highest incidence of parvovirus DNA (78.9%, n = 15 out of 19) (Table 1). In this species, viral DNA was detected among the specimens originated from six out of seven sampled districts (data not shown).

### Absence of Viral Infectious Particles and Autolysis of Tissue Samples may have Hampered Viral Isolation in Parvovirus DNA Positive Samples

Despite detection of short PV DNA fragments enabled by real-time PCR, virus isolation in Candrell feline kidney (CRFK, ATCC CCL#94) cells, attempted with a set of supernatants (n = 11) of tissue homogenates from PCR positive specimens failed in all cases, probably due to the low viral charge in the tissues, as indicated by the high Ct values found during the screening PCR test, and/or the absence of infectious particles.

The mesenteric lymph node and/or intestine of PV-positive animal specimens were under significant autolysis (specifically, 90% of mongoose’s and 74% of red foxes’ tissues, as indicated by microscopic examination), which may also have contributed to unsuccessful virus isolation.

### Detection of Parvovirus Antibodies in Lung Tissue Extracts Confirmed the Circulation of Parvovirus in the Wild

As sera samples were not available in our study, twenty-nine lung tissue extracts (LTEs) prepared from a subgroup of parvovirus DNA-positive animals and ten LTEs from PV DNA-negative specimens were tested by a modified commercial indirect ELISA to detect antibodies from all the animal species included in the survey. This modified ELISA methodology detected CPV/FPLV antibodies among all species, with 26 LTEs (90%) from DNA-positive animals being considered serologically reactive (sample optic density/positive control optic density, S/P>0.150): 18 from mongoose (n = 20, 90%), two from red fox (n = 2), three from Eurasian badger (n = 3), one from stone marten (n = 1) and two from genet (n = 3). The LTEs from two mongooses (S/P>0.7) and one juvenile female badger (S/P = 1.99) from Moura-Barrancos region, one of the last strongholds of the Iberian lynx, were particularly reactive. None of PV DNA-negative specimens had detectable PV antibodies.

### Molecular Characterization of Parvoviruses from different Hosts Based on VP2 Disclosed FPLV-like Viruses in Mongooses and Red Foxes, and CPV-like Virus in Stone Marten

**Figure 3:**
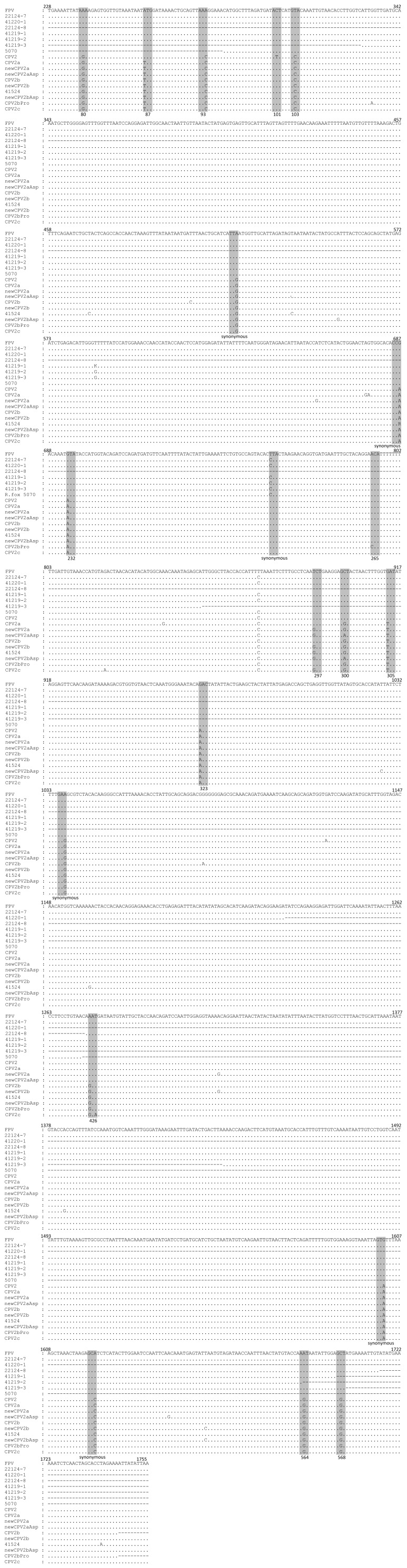
Multiple alignment of VP2 encoding sequences (nucleotides 228 to 1755). CLUSTAL W was used to align *vp2* nucleotide sequences from wild carnivore parvoviruses characterized during this study and from parvoviruses representatives of each virus type. FPLV, on the top line, is represented by the *vp2* sequence from a lion isolate (EF418569). Sequences 22124-7 (JF422105), 41220-1 and 22124-8 (JF422106) were obtained from E. mongooses, while sequences 41219-1, 41219-2 and 41219-3 refer to red fox specimens and sequence 41524 to the stone marten isolate. Other virus types are represented by strains M23255 (CPV-2), DQ340410 (CPV-2a), DQ340422 (newCPV-2a), AB054222 (newCPV-2a-Asp300), AF306450 (CPV-2b), AB054221 (newCPV-2b), AB054224 (newCPV-2b-Asp300), AF306449 (CPV2b-Pro265) and FJ005240 (CPV-2c). Nucleotides which are identical to those in FPLV are represented by dots, while those that differ are indicated. Dashes represent non determined nucleotides. The position of nucleotides within *vp2* is indicated above FPLV reference sequence. Triplets corresponding to characteristic amino acid positions are shaded and their position is indicated below each triplet. Triplets harboring nucleotide variations between feline and canine strains that encode the same amino acid are also shaded and referred as synonymous.

**Figure 4:**
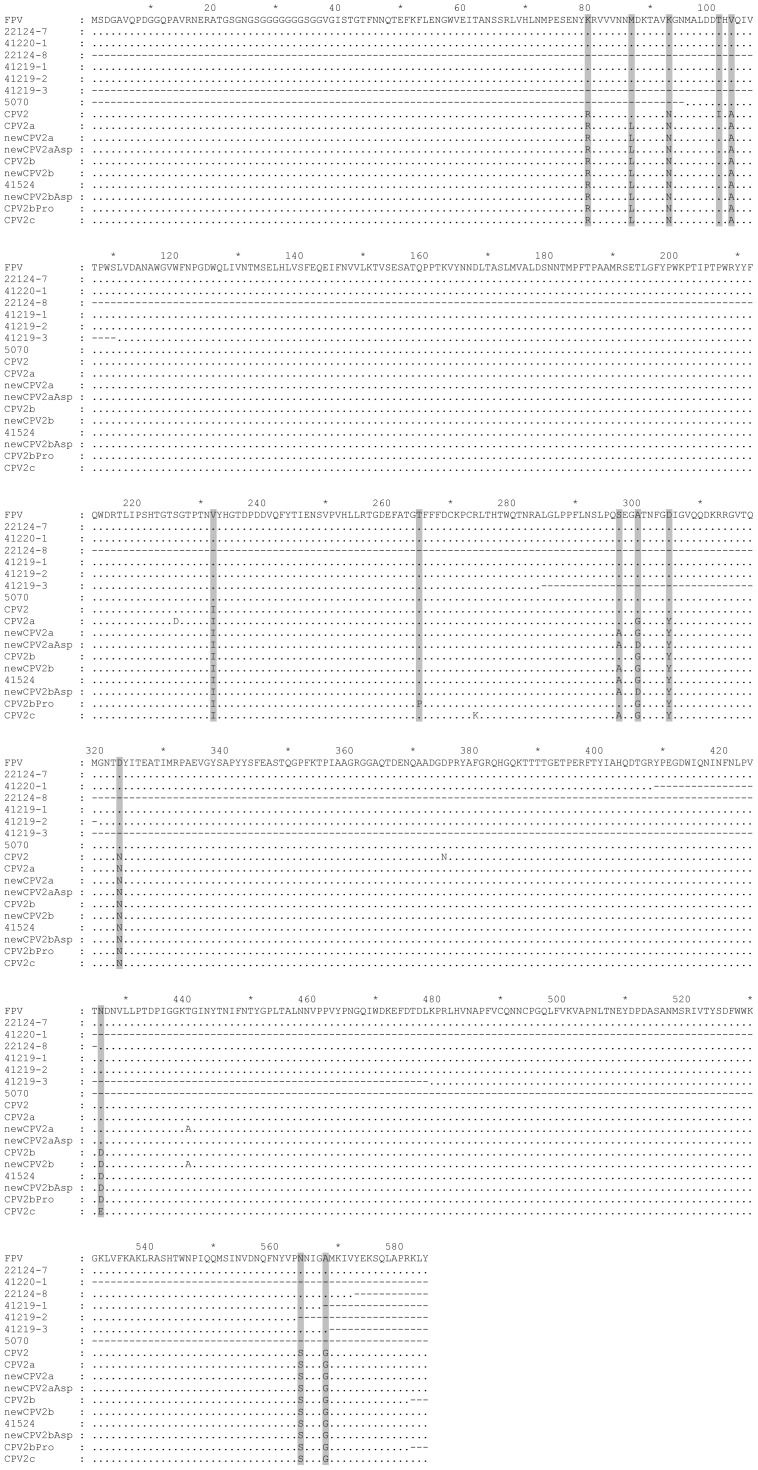
Multiple alignment of VP2 complete amino acid sequences. CLUSTAL W was used to align the deduced VP2 sequences from wild carnivore parvoviruses characterized during this study and from parvoviruses representatives of each virus type. Characteristic amino acid positions are shaded. The amino acid sequences represented correspond to a lion isolate (EF418569), on the top, sequences 22124-7 (JF422105), 41220-1 and 22124-8 (JF422106) to E. mongooses, sequences 41219-1, 41219-2 and 41219-3 to red foxes, and sequence 41524 to the stone marten isolate. Other virus types are represented by strains M23255 (CPV-2), DQ340410 (CPV-2a), DQ340422 (newCPV-2a), AB054222 (newCPV-2a-Asp300), AF306450 (CPV-2b), AB054221 (newCPV-2b), AB054224 (newCPV-2b-Asp300), AF306449 (CPV2b-Pro265) and FJ005240 (CPV-2c).

Without virus isolation, amplification of the complete *vp2* gene (2 kb-long) for molecular characterization of parvoviruses circulating in the animal species surveyed proved difficult and was only fully achieved with viral DNA extracted from one mongoose [specimen 22124-7, captured during 2009 (JF422105)] and one stone marten [specimen 41524, collected during 2010 (JX411926)]. Only partial nucleotide sequences of the *vp2* gene were obtained from tissues of two mongooses [specimens 22124-8 (JF422106) and 41220-1, both obtained during 2010]) and four red foxes [specimens 41219-1, 41219-2, 41219-3 and 5070, all obtained during 2010] (Fig. 3). Polypeptide sequences were deduced and the respective amino acid residues occupying informative positions in VP2 protein were compared with the homologous residues of FPLV and CPV, including CPV-2, CPV-2a, newCPV-2a, CPV-2b, newCPV-2b and CPV-2c variants (Fig. 4).

The complete *vp2* sequence from mongoose 22124-7 (JF422105) and partial *vp2* sequence from mongoose 41220-1 showed all characteristic FPLV amino acid residues [2], indicating that both are FPLV-like viruses (Fig. 3 and Fig. 4). Although only a 442 bp-long sequence was obtained from mongoose 22124-8 (Fig. 3), amino acids found at positions 426, 564 and 568 indicate that this strain is also a FPLV–like virus (Fig. 4). Similarly, the amino acids deduced from partial *vp2* sequences obtained from the four red fox specimens (41219-1, 41219-2, 41219-3 and 5070), showed characteristic FPLV residues at key positions (Fig. 4).

The comparison of the disclosed nucleotide regions within *vp2* from mongoose and red fox FPLV sequences showed that the degree of genetic similarity is very high. In fact, apart from nucleotide position 588, in which T and G were both present in red foxes *vp2*, 100% identity was found among all the homologous sequences analyzed (Fig. 3). Also interesting, the comparison of these FPLV *vp2* sequences with sequences available in *GenBank* evidenced marked similarity with parvoviruses from captive Felidae and domestic cats from Portugal (ranging from 99.94 to 99.88%) [25], followed by FPLV strains from Italy (99.82%).

Amplification of full *vp2* gene for sequencing was only successful with the material from one stone marten specimen among the three that tested PV DNA-positive (JX411926).

Amino acids occupying positions 93 (Asn), 103 (Ala) and 323 (Asn) that are critical for the ability of CPV to replicate in dogs were found preserved in the stone marten parvovirus. Also, CPV-characteristic residues at positions Arg-80, Ser-564 and Gly-568, together with Asp-426 and Ala-297, revealed that the stone marten strain is a newCPV-2b virus type (Fig. 3 and Fig. 4).

### Bayesian Analysis Exposed the Close Genetic Relationship between Mongoose and Large Felidae Strains from Portugal and between Stone Marten and Asian Leopard Cat Strains

**Figure 5:**
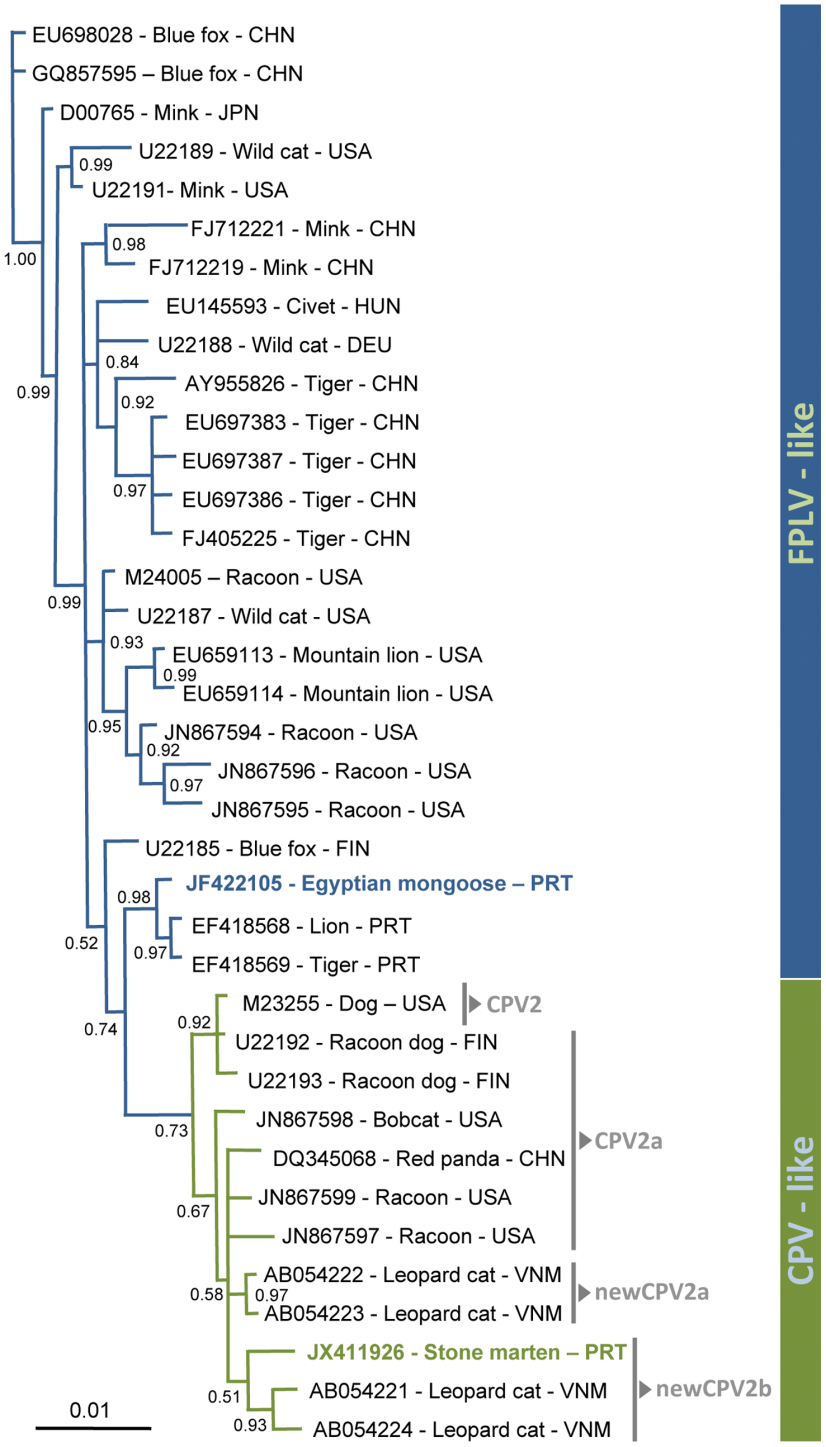
Bayesian Analysis. Bayesian analysis of the *vp2* complete nucleotide sequences from one mongoose and one stone marten obtained during this study and other wild carnivore parvoviruses available in *Genbank* (Table S1). A phylogenetic tree was obtained with a Bayesian inference of phylogeny throughout the MrBayes v3.1.2 software, using the GTR model (nst = 6) with gamma-shaped rate variation with a proportion of invariable sites (rates = invgamma). The analysis was performed with ngen = 10^6^, nchains = 4 and samplefreq = 10. The numbers included on each boot strap represent the Bayesian posterior probability.

Bayesian analysis was performed with two complete *vp2* nucleotide sequences obtained during this study (JF422105 and JX411926) and thirty five *vp2* sequences (25 FPLV-like; ten CPV-like strains) from different wild carnivore species (Table S1, Fig. 5). The stone marten strain from Portugal that was typified as newCPV-2b grouped within the CPV-like cluster, showing the highest genetic proximity with a newCPV-2b leopard strain from Vietnam (Fig. 5).

The mongoose isolate clustered with two FPLV strains from a tiger and a lion obtained during an outbreak that occurred in 2006 at the Lisbon Zoo, Portugal [25] (Fig. 5).

### Modelling Analysis Showed No Influence of Biometric Variables and Sample Origin on Parvovirus Detection in Mongooses

**Figure 6:**
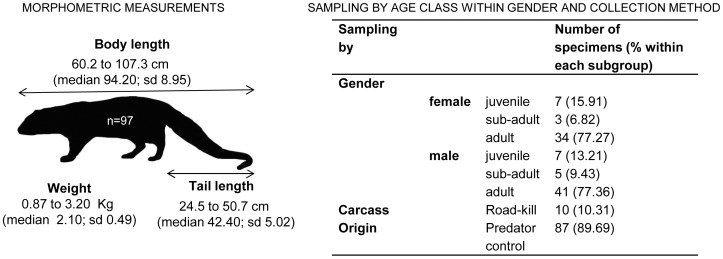
Biometric measurements and collection method of mongoose subpopulation (n = 97) used in modeling analysis.

The influence of gender, age, morphometry and carcass origin on the detection of parvovirus was investigated on PCR positive and negative mongooses (Fig. 6), whose representative sampling (n = 99), enabled a more robust modeling analysis. Two positive specimens were excluded as gender information was not available.

Our data revealed a non-significant spatial autocorrelation of the collected samples (Moran’ I = 0.09; p = 0.206). Two of the morphometric variables were highly correlated (body total length versus tail length: r = 0.773; body mass versus body condition: r = 0.749), and thus tail length and body mass were excluded from further analysis.

A set of 30 models was created and tested and five models were identified as the most parsimonious (AICc<2) to describe the detection of FPLV in this species (Table S2). Those models included the null model together with others combining age, origin and body condition (Table S2). The AUC value, derived from the ROC curve reached 0.609 (sd = 0.057), revealing a low accuracy according to criteria defined elsewhere [26]. Therefore, we conclude that, in this study, the detection of parvovirus in mongooses cannot be predicted by influence of any of the independent variables tested (age class, body condition or corpses origin).

## Discussion

Parvoviruses are considered endemic in most domestic and feral carnivore populations worldwide [1],[2]. The incidence rates detected during this study in widely distributed wild carnivore species from mainland Portugal are consistent with this notion. Although sample sizes were statistically limited, the incidence of parvovirus found in red foxes (78.9%), genets, stone martens and badgers (virtually all specimens tested) is notable. Also remarkable is the rate of parvovirus DNA present among mongooses (58%), which, to our knowledge, is reported for the first time in this species. Even though most specimens presented autolysis, the use of tissues, such as lymph nodes and intestine, as matrices in our methodological approach, together with the higher sensibility of real-time PCR facilitated viral detection and evaluation of parvovirus prevalence in wild species, in comparison with other studies performed in the country relying on the analysis of scats with conventional PCR [19]. The high Ct values registered with autolysed positive samples contrasted with lower Ct values routinely obtained with fresh samples from cats and dogs under active disease (Duarte *et al.*, unpublished results), suggesting that only small amounts of parvovirus DNA were present, likely corresponding to persistent viral DNA left over from previous infections. This possibility explains unsuccessful virus isolation in CRFK cells and the absence of evident macro- or microscopic pathological lesions in the subset of specimens that were not under autolysis. The difficulty found in the amplification of the full *vp2* gene, aiming the molecular characterization of the virus, is also sustained by this hypothesis. Difficulty in viral isolation from feces and intestine samples of wild species, particularly red fox, has been referred before [27],[28].

In agreement with the study of Santos et al. [17], we detected CPV/FPLV antibodies in red foxes, stone martens and genets, as well as in the other species surveyed, with a total of 26 (90%) out of 29 LTEs from DNA-positive animals being serologically reactive. However, to our knowledge, this is the first report on the detection of parvovirus antibodies in badgers. Parvovirus antibodies have been reported in wild red foxes from other geographical areas, namely Spain [29] and Canada [30]. Interestingly, the antibody prevalence observed in Canada was very close to the viral prevalence found in this species during our study (78% out of 19 surveyed animals).

Amplification of complete or even partial *vp2* gene sequences allowing the molecular characterization of the viruses from badgers and genets was not achieved in our study. In contrast, our sequencing results clearly show that the mongoose and red fox strains belong to the feline parvovirus cluster, as they encode key amino acids characteristic of FPLV. Whereas FPLV is maintained independently in the mongoose and red fox populations, or acquired through the direct or indirect contact with other infected wild or domestic carnivores, is unknown. Parvovirus is extremely stable and can remain infectious in the environment for many months, which facilitates transmission by fecal-oral route among susceptible species. Wild species, particularly red foxes and raccoons, have been pointed as candidate evolutionary intermediates of FPLV-CPV [27] and CPV-2-CPV-2a parvovirus evolution [31] respectively. The molecular data obtained during our study, regarding the characterization of parvoviruses from *Vulpes vulpes* in Portugal, does not clarify this hypothesis, as we cannot exclude that domestic cats, or even mongooses, may have been the source of FPLV-like viruses infection to these foxes. The higher degree of genetic similarity between the FPLV viruses from different wild species originated in Portugal, in comparison with the FPLV sequences presently available in *GenBank*, may suggest the geographic clustering of parvoviruses that circulate in Western Iberia. A phylogenetic analysis using the two *vp2* complete sequences obtained during this study and thirty five strains from several wild species reinforces this hypothesis, as the mongoose FPLV-like virus grouped with two FPLV strains from large captive Felidae from Lisbon, diverging only in two nucleotide positions (760 and 871). On the other hand, parvovirus from *Martes foina* was molecularly characterized as new-CPV-2b, providing the first publicly available parvovirus *vp2* nucleotide sequence from this species. Although a stone marten CPV-2a strain was referred by Steinel et al. 2001 [2], its nucleotide sequence is not available in *Genbank*. The phylogenetic analysis showed a close genetic similarity of the stone marten isolate with Asian leopard parvoviruses, also belonging to the newCPV-2b subtype. Due to the genetic similarity found between the stone marten *vp2* sequence and a newCPV-2b domestic dog strain from USA (EU659121), and the circulation of different CPV-2b strains in Portugal [32], it is likely that the stone marten virus may have been originated in infected domestic species, and do not appear to represent an evolutionary intermediate in the CPV-2b branch.

Parvoviruses have been isolated from the feces of clinically healthy domestic and wild felines [33]. Although a carrier stage has not been defined for dogs after infection by CPV, it has been observed that asymptomatic adult dogs may periodically shed the virus [34]. It is also known that infected cats, whether symptomatic or not, serve as reservoirs and source of infection to other animals [35]. Concerning the animal species under study, data on the severity of the disease has been reported for red foxes experimentally infected [30]. In that study, a marked immune response was apparently developed after infection with FPLV but no clinical signs were detected with MEV and CPV-2 inoculation [30]. Regarding previous works in badgers, macro and microscopic lesions suggestive of CPV myocarditis on an adult specimen have been reported [36], as well as parvovirus-associated enteritis followed by death of badger cubs [37]. Our work was inconclusive with this respect, as tissue autolysis of positive badger specimens limited the investigation of pathological findings. To our knowledge, there are no virological reports dedicated to the study of the susceptibility of herpestids, particularly the Egyptian mongoose, to parvovirus infection. However, Millan and collaborators (2009) have previously reported the detection of parvovirus antibodies in this species (n = 18, 50%) [38]. The remarkable prevalence found during this study in red foxes and mongooses, together with the concomitant lack of evident gross gastro-enteric lesions during necropsy, argues for the presence of viral DNA persisting from previous infections. Any significant parvovirus disease is likely in neonatal or young animals, so the lack of lesions suggestive of acute parvovirus infection at necropsy would likely not be connected to the effects of the original infections by these viruses on the specimens surveyed. Furthermore, as sampling did not include animals found naturally dead, or apparently sick, the possibility that mongoose or red fox may also undergo acute disease cannot be overlooked. No specimen had clinical signs suggestive of haemorrhagic enteritis or congestion, as observed in cats or dogs under active disease. In addition, whenever present, faeces were consistently moulded and no signs of diarrhoea were ever noticed.

The lack of knowledge on the susceptibility, pathogenicity and morbidity of the FPLV/CPV infection in many wild species, including mongoose, genet and stone marten hampers the true understanding of the dynamics of this disease in wild populations. Some studies have evaluated the factors influencing the seroprevalence of viral agents in wildlife and showed a clear influence of age on the exposure risk [39]. However, in our modelling effort, we failed to detect such influence on mongoose susceptibility to parvovirus. The mongoose population that we surveyed was strongly biased by adult specimens. Thus, we believe that other factors, not considered in our study due to lack of data, such as domestic pet distribution in rural areas (e.g. free ranging domestic cats), and species-specific ecological variables, like feeding habits and land use, or carnivore guild diversity, distribution, and social character, may be crucial in the patterns of infection of wild species by this viral agent.

The significant exposure of wild carnivores to parvovirus, particularly in certain geographical regions of mainland Portugal that include sensitive areas for conservation (e.g. along the Guadiana river and nearby the Malcata Mountain) is of relevance, since epizootics may lead to declines in infected populations [2],[38],[40]. This is especially dramatic if species of conservation value or endangered are affected, as is the case of the Iberian lynx (*Lynx pardinus*), the most endangered felid in the world [38] that is expected to be reintroduced in Portugal in the future. The introduction of parvovirus-susceptible lynx [38] into geographical areas where the virus is circulating in the wild, and potentially excreted to the environment, should take into account the effective risk of transmission to the particularly susceptible young cubs. Targeted surveillance efforts should therefore be reinforced to improve our understanding of the ecological and epidemiological factors associated with microbial infection, in order to develop sustained management and conservation strategies for animal species at risk.

## Materials and Methods

### Animal Samples Collection and Study Area

One hundred and twenty eight animals from the Herpestidae, Canidae, Mustelidae, and Viverridae families of the order Carnivora, including Egyptian mongoose (*Herpestes ichneumon*, n = 99), red fox (*Vulpes vulpes*, n = 19), Eurasian badger (*Meles meles*, n = 4), stone marten (*Martes foina*, n = 3) and common genet (*Genetta genetta*, n = 3), were analyzed in this study. The complete panel of viruses tested is described below and also indicated in Table 1. Samples, collected from 2008 to 2011, included animals from road-kills (n = 26, all surveyed species) and animals captured under legal game management actions aiming the control of predator densities [mongoose (n = 93) and red fox (n = 9)].

No animals were sacrificed for the purposes of this specific study. None of the authors were responsible for the death of any animals nor were any samples used in the study collected by the authors. Road killed animals were collected by the road maintenance technicians of “EP - Estradas de Portugal, S.A”, whenever they were found accidentally dead as the result of vehicle-wildlife collisions in the roads under surveillance and management by the company. This organized animal sample collection and donation results from a collaboration protocol established between CBA/FCUL (Universidade de Lisboa, Centro de Biologia Ambiental, Faculdade de Ciências de Lisboa) and “EP - Estradas de Portugal, S.A”, which is entitled " Monitoring of vertebrate mortality caused by road-kill in Portuguese roads", which aimed to update carnivore distribution maps, genetically characterize some predator species and to model carnivore habitat connectivity in a conservation perspective.

Carnivores hunted in the scope of hunting activities or predator control actions, legally authorized by the National Forest Authority (*Autoridade Florestal Nacional*) that emits permits for those actions, were gathered by hunting associations. Animals were killed in legal hunting sessions (following the Portuguese game legislation) by hunters with valid permits assigned by “Autoridade Florestal Nacional”, and totally or partially donated for scientific purposes by the hunting associations/confederations responsible for managing the hunting journeys.

Shortly after death, or shortly after being found dead on the road, animal carcasses were preserved in sealed plastic bags, taken refrigerated into a collection center and kept frozen at −20°C until necropsy. Animal Corpse transportation was done under the license from the Institute for Nature Conservation and Biodiversity (ICNB – Licence no. 222/2010/TRANS).

Animals were sampled from 15 out of the 18 administrative regions (districts) of mainland Portugal, the westernmost country of the Eurasian supercontinent (Fig. 1A). Geographical location coordinates and date of collection of all sampled animals were recorded. The majority of the animal specimens were sexed and age was determined according to tooth characteristics and size. Age was not available for six animals while sex was not recorded for 14 specimens. Adults were predominant (76.5%). The subpopulation for which sex data was available (89%) exhibited a similar predominance of males (51.75%) and females (48.25%). Samples from lung, spleen, liver, small intestine and mesenteric lymph node were collected from each animal during necropsy and processed for further analysis. All animals showing clear signs of putrefaction were excluded from this analysis. No gross lesions were apparent at autopsy, even though histopathological examinations evidenced autolysis in, approximately, 90% of specimens.

### Detection of Viral Pathogens

The presence of Parvovirus (PV), Coronavirus (CoV), Canine Distemper Virus (CDV), Feline Herpesvirus (FHV), Aujeszky Disease virus (ADV), Canine Adenovirus types 1 and 2 (CAV 1 and CAV2) and Influenza virus (IV) was preliminarily investigated in wild carnivores for a subset of animal specimens (n = 34), using the tissue matrices and following the methods indicated in Table 2 [41],[42],[43],[44],[45]. Subsequently, the detection of parvovirus in mesenteric lymph nodes and small intestine tissue samples was attempted for all animal specimens (n = 128) using the molecular technique described by [46] that targets a 93 bp-sequence of the *vp2* gene (Tables 2 and 3).

**Table 2:** Methods, tissues and genomic regions used for virus detection.

Viruses	Nucleic acid	Kit used for amplification	Type ofPCR	Genomic region Targeted	Size of the amplicon	Tissue	Reference
CPV/FPLV	DNA	Fast start Master Mix, Roche	*real-time*	*vp2* gene	93 bp	small intestine lymph nodes	[46]
FCoV/CCoV	RNA	One-step RT-PCR, Qiagen	*real-time*	*7b* gene	102 bp	small intestine lymph nodes	[41]
CDV	RNA	One-step RT-PCR, Qiagen	*real-time*	*N* gene	161 bp	lungs	*in house* (not published)
ADV	DNA	Fast start Master Mix, Roche	*real-time*	*gB* gene	94 bp	lungs	[42]
CAV-1	DNA	High Fidelity Master Mix, Roche	*conventional*	*E3* gene	508 bp	liver	[43]
CAV-2	DNA	High Fidelity Master Mix, Roche	*conventional*	*E3* gene	1030 bp	lungs	[43]
FHV	DNA	High Fidelity Master Mix, Roche	*real-time*	*TK* gene	56 bp	lungs	[44]
Influenza A	RNA	One-step RT-PCR, Qiagen	*real-time*	Matrix gene	100 bp	lungs	[45]

**Table 3:** Primer sequences and their positions in the genome of parvovirus.

Primer	Nucleotide sequence (5′–3′)	Position^a^	Sense	Specificity	Ref.	Purpose of use
CPV-For	AAACAGGAATTAACTATACTAATATATTTA	4101–4130	+	FPLV&CPV^m^	[46]	Viral survey
CPV-Rev	AAATTTGACCATTTGGATAAACT	4138–4167	−	FPLV &CPV		
CPV-Probe	FAM-TGGTCCTTTAACTGCATTAAATAATGTACC-TAMRA	4171–4193	+	FPLV &CPV		
CPV-1F	ACCAGATCATCCATCAACATC	2653–2673	+	FPLV &CPV	[51]	*vp2* gene amplification
CPV-1R	CAATTAGTTGCCAATCTCCTG	3153–3173	−	FPLV &CPV		
CPV-2F	AAATTGTAACACCTTGGTCATTG	3096–3118	+	FPLV &CPV		
CPV-2R	AAATGGTGGTAAGCCCAATG	3636–3655	−	FPLV &CPV		
CPV-3F	ACAGGTGATGAATTTGCTACAG	3554–3575	+	FPLV &CPV		
CPV-3R	TTACAGGAAGGTTAAAGTTAAT	4037–4058	−	FPLV &CPV		
CPV-4F	CAACAGGAGAAACACCTGAG	3954–3974	+	FPLV&CPV^m^		
CPV-4R	TCTTCTATTTCTTACAGTTATTG	4718–4740	−	FPLV &CPV		
555-For	CAGGAAGATATCCAGAAGGA	4002–4021	+	FPLV &CPV	[52]	*vp2* gene amplification
555-Rev	GGTGCTAGTTGATATGTAATAAACA	4561–4585	−	FPLV &CPV		
P1	ATGAGTGATGGAGCAGTTC	2786–2804	+	FPLV &CPV	[12],[50]	*vp2* gene amplification
PR	TTTCTAGGTGCTAGTTGAG	4512–4530	−	FPLV &CPV		

a position in the complete genome of strain CPV-N (NC001539). ^m^mismatch in some isolates.

The appropriate tissues collected during necropsy (Table 2) were homogenized with PBS and submitted to nucleic acids extraction in a BioSprint 96 nucleic acid extractor (Qiagen, Hilden, Germany), according to the manufacturer’s protocol. Extensive DNA/RNA contamination precautions were taken during all stages of experimental work to avoid DNA/RNA carry-over.

The presence of viral DNA and viral RNA was tested using the FastStart TaqMan Probe Master (Roche, Roche Diagnostics GmbH, Mannheim, Germany) and the OneStep RT-PCR (Qiagen, Hilden, Germany) commercial kits, according to Table 2. Six tissue samples testing positive for parvovirus and the coronavirus positive sample were re-tested using different amounts (2.5, 5.0, 7.5 and 10 µL) of DNA and RNA, respectively, to determine the more appropriate volume to be used in the subsequent viral screenings. The lowest Ct values were obtained, respectively with 5 µL of DNA and 10 µL of RNA per PCR reaction, although all the other volumes generated positive signals. Based on the results obtained, aliquots of 5 µL of DNA or 10 µL of RNA were added to each 25 µL PCR reaction containing 1 µM of each PCR primer (Eurofins MWG Operon, Ebersberg, Germany) and 0.2 µM of the TaqMan probe (Eurofins MWG Operon, Ebersberg, Germany). Amplifications were done in a Bio-Rad CFX96™ Thermal Cycler (Bio-Rad Laboratories Srl, Redmond, USA) taking into account the annealing temperatures of the primers and the characteristics of the amplification kits used for each nucleic acid amplification. For parvovirus amplification, reactions were carried out at 95°C for 10 min, followed by 40 cycles of 95°C for 15 s, 52°C for 30 s and 60°C for 30 s, while, for coronavirus amplification, reactions were composed by a step of 30 min at 50°C, followed by 15 min at 95°C and 50 cycles of 95°C for 15 s, 56°C for 30 s, 72°C for 30 s. For the remaining viral agents, PCR conditions were those described at the references enlisted in Table 2. Primers and probes used for parvovirus detection and characterization are described in Table 3 **.** Positive and negative controls were used in each assay. Fluorescence measurements were recorded after each annealing step. Data was analyzed with the appropriate sequence detector software (*Bio-Rad CFX Manager*, version 3.0). The specificity of the primers and probe used in this survey for parvovirus DNA detection [46] was assessed against the NCBI nucleotide sequence database (27.11. 2011) (ftp://ftp.ncbi.nih.gov/blast/db/FASTA). The *in silico* analysis showed 100% identity between the nucleotide sequences of the primers and probe and the homologous sequences in Canine, Feline and Mink parvoviruses, demonstrating that this real-time PCR, published as a molecular tool to detect and quantify CPV in dog samples [46], detects also Feline parvoviruses.

Non-template controls were included in each assay to rule out contamination within the PCR reagents. A commercial vaccine containing the old CPV-2 strain (Primadog, Merial, France) was used in each assay as positive control. For all calculations, the baseline was set automatically and the fluorescence threshold manually. The negative controls never crossed the threshold line in any of the experiments, appearing as horizontal flat lines, while the positive control confirmed the performance of the PCR. A number of positive samples were retested by PCR without the inclusion of positive control. Consistency in the generated results ruled out contamination at sample level.

### Parvovirus Isolation

Parvovirus isolation was attempted with a number of tissue PCR-positive samples, specifically those displaying low Ct values (five mongooses, two red foxes, one stone marten, and three genets). Lymph node and intestinal samples were homogenized and suspended in phosphate-buffered saline containing penicillin, streptomycin and amphotericin B (antibiotic-antimycotic), used according to the manufacturer (Gibco, Life Technologies Corporation, Carlsbad, USA). Following centrifugation, the supernatant was filtered using a 0.45 µm filter and used to inoculate subconfluent Crandell feline kidney (CRFK, ATCC CCL#94) cell monolayers, grown in Dulbecco’s modified Eagle’s medium supplemented with 8% FCS (Gibco, Life Technologies Corporation, Carlsbad, USA) and 50 µg/ml gentamycin (Gibco, Life Technologies Corporation, Carlsbad, USA). Cell cultures were observed daily for cytopathogenic effect (CPE). After 5 days, cultures with no apparent CPE were trypsinized and maintained for further 5 days. Failure in virus isolation was considered after four passages with no CPE and negative Parvovirus-PCR of the culture supernatants.

### Serological Assays

Lung tissue extracts (LTE) were prepared as described by Ferroglio et al. [47] and used to test the presence of circulating antibodies against parvovirus in PCR positive specimens. Briefly, lung tissue was homogenized in PBS, centrifuged at 10000xg for 10 min and the supernatant conserved at −20°C. The presence of antibodies against parvovirus (feline or canine parvoviruses) in LTEs was determined with a commercial indirect ELISA test for canine parvovirus antibodies (Ingezim CPV© 15.CPV.K1, Ingenasa, Madrid, Spain). Using this ELISA test, it is not possible to determine if the antibodies detected are raised against FPLV or CPV due to the high antigenic similarity between these viruses. In order to detect antibodies from the different carnivore species, the anti-dog conjugate was replaced by Protein A-Peroxidase *Staphylococcus aureus*/horseradish (PA-HRPO) (Sigma-Aldrich, St. Louis, USA) at the dilution of 1∶2000, determined after titration of PA-HRPO in parallel with the kit conjugate, which generated OD values for the positive and negative controls identical to those obtained with the kit conjugate. All the other steps followed the recommendations of the manufacturer. This adapted ELISA has been used successfully in our laboratory to detect parvovirus antibodies in serum samples of large Felidae, namely lions and tigers (Duarte et al., unpublished results). LTEs of seven mongooses and three red foxes that were negative in the real-time PCR were selected as putative antibody negative samples and tested in this adapted ELISA to infer about possible unspecific backgrounds and to evaluate the suitability of the kit cut off value for the ELISA PA-HRPO. Lower non-specific reactivity was detected in LTEs from PCR negative samples when using protein A-HRPO. The OD_450 nm_ values obtained in mongoose’s LTEs ranged from 0.057 to 0.135, corresponding to a S/P ratio (sample optic density/positive control optic density) of 0.03 and 0.07, respectively, and in red-fox’s LTEs ranged from 0.060 to 0.101, corresponding to a S/P ratio of 0.05 and 0.04, respectively. These results confirmed the successful use of protein A-HRPO in ELISA to detect mongoose IgGs. Protein-A conjugate has also been used in other studies to detect fox IgGs [48],[49]. The cut-off established by adding 2 standard deviations (SD) to the mean of the negative control duplicates was in agreement with the cut-off criteria of the kit (samples considered positive if S/P value above 0.150). Therefore, the validation criteria of the test were adopted: test was considered valid if OD_450nm_ of the positive control>1.0 and OD_450 nm_ of the negative control<0.150. For interpretation of the ELISA PA–HRPO test results, samples were considered positive if S/P value was above 0.150. To evaluate the sensibility of the modified test, LTEs from two foxes, that were found respectively antibody positive and antibody negative by this adapted ELISA, were also tested in parallel with the heterologous conjugate (anti-dog IgG-HRPO) from the commercial ELISA, since sufficient cross-reactivity exist between anti-canine IgG and serum antibody from red foxes [49]. Our results showed that the ELISA using protein A affinity conjugate was slightly less sensitive for measuring anti-parvovirus antibodies in foxes than the equivalent commercial ELISA. In the case of the antibody positive red fox LTE, S/P ratios of 0.304 and 0.429 were obtained with the modified and commercial ELISA test, respectively; and, in the case of the fox LTE testing antibody negative, ratios of 0.03 and 0.064 were registered, respectively.

### Molecular Characterization of Parvovirus

The molecular characterization of parvovirus from ten mongooses, three common genets, three badgers, three stone martens and eight red foxes was endeavored through amplification of the complete *vp2* gene (approximately 1.9 kb), attempted by using different combinations of primers described by [25],[50],[51],[52] (Table 3).

Amplification was carried out with the High Fidelity PCR Master Mix (*Roche Diagnostics GmbH*, Mannheim, Germany), by performing 50 cycles of denaturation at 94°C for 30 s, annealing at 50°C for 30 s and extension at 72°C for 2 min, followed by a final extension step of 10 min at 72°C. After electrophoresis on a 1.5% agarose gel with red gel staining (GelRed Nucleic acid stain, Biotium), the PCR products were excised and purified by a commercial kit (QIAquick gel extraction kit; *Qiagen,* Hilden, Germany). The resulting products were directly sequenced using a 3130 Genetic Analyser (*Applied Biosystems*, Foster City, CA, U.S.A). Sequences were analyzed using *Seqscape* software v2.7 (Applied Biosystems, Foster City, CA, USA) and the polypeptide sequences deduced in order to determine and differentiate the type of virus present.

### Phylogenetic Analysis

For Bayesian analysis, the *vp2* complete nucleotide sequences from one mongoose (Herpestidae) and one stone marten (Mustelidae) obtained during this study (JF422105 and JX411926) were compared with the *vp2* sequences from several wild carnivore parvoviruses available in the NCBI database (Table S1), namely felids (tiger, wildcat, lion, mountain lion, leopard, lynx), viverrids (civet), mustelids (mink), canids (blue fox, raccoon dog), procyonids (raccoon), and ailurids (red panda). A PPV (pig parvovirus) isolate was used as an outer group.

Multiple alignments were generated by CLUSTAL W [53] and the result was converted to the NEXUS format using Mesquite software [54]. The phylogenetic tree was obtained with a Bayesian inference of phylogeny throughout the MrBayes v3.1.2 software that uses Markov chain Monte Carlo simulation technique to approximate the posterior probabilities of trees [55],[56]. MrBayes analysis was performed using the GTR model (nst = 6) with gamma-shaped rate variation with a proportion of invariable sites (rates = invgamma). The analysis was run for 10^6^ generations (ngen = 10^6^) with four chains of temperature (nchains = 4) and each chain was sampled every 10^th^ generations (samplefreq = 10).

### Analysis of the Influence of Biometric Variables and Sample Origin on Parvovirus Detection in Mongooses

The influence of gender, age class (juvenile, subadult, and adult), morphometry (body total length, tail length, body mass and body condition) and corpse’s origin (road-kill versus predator control) on parvovirus positivity (detection/non-detection) was tested by using a Generalised Linear Model (GLM) with a binomial distribution and a logit link function [57]. With the exception of the body condition index, all morphometric measures were taken during necropsy. The residuals from ordinary least squares (OLS) regression of body mass on total length were used as indices of body condition [58].

Prior to the modeling procedure, spatial autocorrelation was evaluated by the Moran’ I index [59] to assure no violation of the assumption of independence, with the consequent pseudoreplication [60],[61]. Multicollinearity between morphometric variables was also tested using the Spearman’s rank correlation coefficient (r_s_) [62]. For pairs of variables where the correlation was higher than 0.7 [63], the one with a higher biological/ecological meaning was selected.

A set of models was created, corresponding to all combinations of the considered independent variables (gender, age class, and species). To select the most parsimonious model, an information criterion model selection procedure (Akaike’s information criterion for small samples AICc - [64]) was used. The best models were assumed to be those with the lowest AICc value. However, as suggested by [64], those with ΔAICc <2 (ΔAICc being the difference between the AICc of the i^th^ model and the minimum AICc value) were considered best models (Table S2). Finally, the Akaike weights (wi) were calculated to obtain each model’s probability of being the best model for the data [65]. The area under each curve (AUC), derived from receiver-operating characteristics plots (ROC), was used to evaluate the performance of the model [26].

All modelling analyses were performed using R software, version 2.10.0 (R Development Core Team 2008), together with the “ape - Analyses of Phylogenetics and Evolution” [66] and the MuMIn [67] packages.

## Supporting Information

Table S1 **Information on the complete** ***vp2*** **nucleotide sequences used for the phylogenetic analysis of wild carnivore parvovirus.**
(DOCX)

Table S2 **Summary of best fitted models information criteria.**
(DOCX)
