# Supplementary material for: Snapshot of Viral Infections in Wild Carnivores Reveals Ubiquity of Parvovirus and Susceptibility of Egyptian Mongoose to Feline Panleukopenia Virus
Source: PLoS One. 2013 Mar 20;8(3):e59399. doi: 10.1371/journal.pone.0059399 (PMC3603882; doi:10.1371/journal.pone.0059399)
Supplement: Table S1 — Information on the complete vp2 nucleotide sequences used for the phylogenetic analysis of wild carnivore parvovirus. (DOCX) [file pone.0059399.s001.docx]

**Supporting information**

**Table S1.** **Information on the complete *vp2* nucleotide sequences used for the phylogenetic analysis of wild carnivore parvoviruses**

| ***Genbank* ACCESS NUMBER** | **COMMON NAME** | **SPECIES** | **VIRUS** | **YEAR OF ISOLATION** | **GEOGRAPHIC ORIGIN** | **STRAIN** |
| --- | --- | --- | --- | --- | --- | --- |
| JF422105 | Egyptian mongoose | *Herpestes ichneumon* | FPLV | 2009 | Portugal, Castelo Branco | FPLV/Egyptian mongoose/PT2009 |
| U22185 | Blue fox | *Alopex lagopus* | BFPV | 1983 | Finland | BFPV-1 |
| EU698028 | Blue fox | *Alopex lagopus* | BFPV | 2007 | China | Tai`an |
| GQ857595 | Blue fox | *Alopex lagopus* | BFPV | 2008 | China | BFPV |
| EF418568 | Captive Lion | *Panthera leo* | FPLV | 2006 | Portugal | FPLV/Lion/PT06 |
| EU659113 | Mountain lion | *Puma concolor* | FPLV | 1989 | USA | FPV-8a.us_89 |
| EU659114 | Mountain lion | *Puma concolor* | FPLV | 1989 | USA | FPV-8b.us_89 |
| EU697386 | Tiger | *Panthera tigris* | FPLV | 2007 | China | HT-374 |
| EU697383 | Tiger | *Panthera tigris* | FPLV | 2007 | China | HT-262 |
| EU697387 | Tiger | *Panthera tigris* | FPLV | 2007 | China | HT-163 |
| FJ405225 | Tiger | *Panthera tigris* | FPLV | 2008 | China | - |
| EF418569 | Tiger | *Panthera tigris* | FPLV | 2006 | Portugal | FPLV/Tiger/PT06 |
| AY955826 | Tiger | *Panthera tigris* | FPLV | ≤2005 | China | GT-2 |
| EU145593 | Asian palm civet | *Paradoxurus hermaphroditus* | FPLV | 2007 | Hungary | 389/07 |
| U22189 | Wild cat | *Felis sylvestris* | FPLV | ~1964 | USA | FPV-d |
| U22188 | Wild cat | *Felis sylvestris* | FPLV | 1993 | Germany | FPV-377 |
| U22187 | Wild cat | *Felis sylvestris* | FPLV | 1990 | USA | FPV-23 |
| U22191 | Mink | *Mustela lutreola* | MEV | 1965 | USA | MEV-e |
| D00765 | Mink | *Mustela lutreola* | MEV | 1978 | Japan | MEV-Abashiri |
| FJ712221 | Mink | *Mustela lutreola* | MEV | 2008 | China | LYT-2 |
| FJ712219 | Mink | *Mustela lutreola* | MEV | 2008 | China | Manzhouli |
| JN867596 | Raccoon | *Procyon lotor* | RPV | 1978 | USA,Texas | FPV/Raccoon/TX/Rac1.2/78 |
| M24005 | Raccoon | *Procyon lotor* | RPV | 1979 | USA, Arkansas | - |
| JN867594 | Raccoon | *Procyon lotor* | RPV | 1990 | USA, New Jersey | FPV/Raccoon/NJ/RPV-6/90 |
| JN867595 | Raccoon | *Procyon lotor* | RPV | 1978 | USA, Texas | FPV/Raccoon/TX/Rac2.2/78 |
| JN867596 | Raccoon | *Procyon lotor* | RPV | 1978 | Texas | FPV/Raccoon/TX/Rac1.2/78 |
| JN867597 | Raccoon | *Procyon lotor* | RPV,CPV-2a | 2010 | USA, New York | CPV/Raccoon/NY/94742/10 |
| JN867598 | Bobcat | *Lynx rufus* | RPV, CPV-2a | 2010 | USA, Kansas | CPV/Bobcat/KS/44/10 |
| JN867599 | Raccoon | *Procyon lotor* | RPV, CPV-2a | 2009 | USA, Kentucky | CPV/Raccoon/KY/39552/09 |
| JX411926 | Stone marten | *Martes foina* | NewCPV-2b | 2010 | Portugal, Viseu | CPV/Stone marten/PT2010 |
| AB054221 | Leopard cat | *Felis bengalensis* | LCPV newCPV-2b | 1997 | Vietnam | LCPV V204 |
| AB054222 | Leopard cat | *Felis bengalensis* | LCPV new CPV-2a, Asp300 | 1997 | Vietnam | LCPV V139 |
| AB054223 | Leopard cat | *Felis bengalensis* | LCPV newCPV-2a, Asp300 | 1997 | Vietnam | LCPV V140 |
| AB054224 | Leopard cat | *Felis bengalensis* | LCPV newCPV-2b, Asp300 | 1997 | Vietnam | LCPV V203 |
| U22193 | Raccoon dog | *Nyctereutes procyonoides* | RDP,V CPV-2a, Ala300 | 1978 | Finland | RD-87 |
| U22192 | Raccoon dog | *Nyctereutes procyonoides* | RDPV, CPV-2a, Ala300 | 1980 | Finland | RD-80 |
| DQ345068 | Red panda | *Ailurus fulgens* | RPPV, CPV-2a | 2004 | China | RPPV |
| L23427 | Domestic pig | *Sus scrofa* | PPV | - | - | NADL-2 |
