# Supplementary material for: Snapshot of Viral Infections in Wild Carnivores Reveals Ubiquity of Parvovirus and Susceptibility of Egyptian Mongoose to Feline Panleukopenia Virus
Source: PLoS One. 2013 Mar 20;8(3):e59399. doi: 10.1371/journal.pone.0059399 (PMC3603882; doi:10.1371/journal.pone.0059399)
Supplement: Table S2 — Summary of best fitted models information criteria. (DOCX) [file pone.0059399.s002.docx]

**Supporting information**

**Table S2.** **Summary of best fitted models information criteria**

| Models (variables included) | AICc^1^ | ΔAICc^2^ | AICcwi^3^ |
| --- | --- | --- | --- |
| Null | 134.80 | 0 | 0.124 |
| body_c^4^ | 135.21 | 0.41 | 0.101 |
| c_orig+body_c | 135.26 | 0.46 | 0.099 |
| c_orig^4^ | 135.63 | 0.83 | 0.082 |
| Age | 136.65 | 1.85 | 0.049 |

^1^AICc - Akaike's information criterion.

^2^ΔAICc - difference to the lowest AICc value.

^3^wi - Akaike weights.

^4^body_c - body condition; c_orig - corpse origin.
